# Dose- and state-dependent enhancement of hippocampal CA1 pyramidal neuron excitability by GM1 ganglioside in male rats with cholinergic lesions

**DOI:** 10.1038/s41598-026-58002-2

**Published:** 2026-06-15

**Authors:** Sedigheh Ashkavandi, Ahmad Ali Moazedi, Saeed Semnanian, Saeedeh Abdolahpour, Maryam Gholami

**Affiliations:** 1 Department of Biology, Faculty of Science, Shahid Chamran University of Ahvaz, Ahvaz, Iran; 2 Department of Physiology, Tarbiat Modares University, Tehran, Iran; 3 Growth and Development Research Center, Tehran University of Medical Sciences, Tehran, Iran; 4 Department of Cell and Molecular Biology, School of Biology, College of Science, University of Tehran, Tehran, Iran; 5 Toxicology and Poisoning Research Center, Tehran University of Medical Sciences, Tehran, Iran

**Keywords:** GM1 ganglioside, Alzheimer’s disease, CA1 pyramidal neurons, Single-unit recording, Cholinergic lesion, Neuronal excitability, Neurology, Neuroscience

## Abstract

Cholinergic degeneration contributes to hippocampal circuit dysfunction in Alzheimer’s disease (AD). Ganglioside GM1 modulates membrane signaling and synaptic function, yet its acute effects on hippocampal neuronal activity under cholinergic-deficient conditions remain unclear. Using in vivo single-unit recordings in urethane-anesthetized male rats, extracellular single-unit activity was recorded from CA1 pyramidal neurons before and after systemic GM1 administration. We examined whether systemic GM1 alters spontaneous firing of CA1 pyramidal neurons in a nucleus basalis magnocellularis (NBM) lesion model of cholinergic hypofunction. GM1 (2, 5, 10 mg/kg, i.p.) produced dose-dependent increases in firing rate. Linear mixed-effects modeling, accounting for multiple neurons per animal, revealed a significant main effect of Time (F_1,79_ = 41.33, *p* < 0.001, partial η^2^ = 0.34) and a significant Dose × Time interaction (F_3,79_ = 3.28, *p* = 0.025, partial η^2^ = 0.11). Notably, NBM-lesioned animals exhibited amplified responses, particularly at 2 mg/kg (Δ = 7.22 ± 1.82 Hz), whereas control animals showed minimal change at this dose. GM1-induced increases remained within reported physiological firing ranges under urethane anesthesia. These findings demonstrate that GM1 enhances hippocampal CA1 excitability in a dose- and state-dependent manner, with heightened responsiveness in cholinergic-compromised networks. The results support the concept that membrane-targeting interventions may modulate dysfunctional circuits depending on baseline network state.

## Introduction

Alzheimer’s disease (AD) is a progressive neurodegenerative disorder and a leading cause of dementia worldwide^1^. The hippocampal formation, particularly the CA1 subregion, is critically involved in memory processes and is among the earliest and most severely affected brain areas in AD^2^. Dysfunction within hippocampal networks is a core pathophysiological feature contributing to the cognitive deficits observed in AD^3^.

In addition to structural degeneration, functional alterations in neuronal excitability have emerged as key contributors to AD’s progression. Both clinical and experimental studies indicate that hippocampal and cortical circuits may exhibit abnormal activity patterns, including hyperexcitability during early disease stages, which can lead to network instability and cognitive impairment. Importantly, pharmacological modulation of neuronal firing does not necessarily represent detrimental enhancement; rather, therapeutic interventions may normalize aberrant activity patterns toward physiological ranges depending on baseline network conditions^4–6^.

A key pathological hallmark of AD is the degeneration of cholinergic neurons originating from the basal forebrain^7^. This cholinergic deficit disrupts cortical and hippocampal function, impairing synaptic plasticity mechanisms essential for learning and memory^8^. The nucleus basalis magnocellularis (NBM) lesion model in rodents effectively recapitulates this cholinergic hypofunction, leading to hippocampal network impairments and cognitive deficits that mirror aspects of human AD^9^.

Degeneration of basal forebrain cholinergic neurons produces substantial alterations in hippocampal network dynamics and neuronal responsiveness. Cholinergic projections regulate pyramidal neuron excitability, synaptic plasticity, and oscillatory activity via muscarinic and nicotinic receptor mechanisms. Consequently, cholinergic dysfunction may contribute to both hypo- and hyperexcitable neuronal states depending on circuit context. Therefore, pharmacological agents capable of modulating membrane properties and synaptic responsiveness, such as GM1, may indirectly compensate for cholinergic deficits and contribute to restoration of neuronal activity balance^10,11^.

Despite extensive research, current therapies for AD remain largely symptomatic and fail to adequately address underlying circuit-level dysfunction^12^. There is a pressing need for interventions that directly target and modulate dysfunctional neural networks^13^. GM1 ganglioside, a monosialoganglioside highly enriched in neuronal membranes, has emerged as a promising candidate due to its multifaceted roles in membrane dynamics and signal transduction^14,15^.

GM1 integrates into lipid rafts, forming dynamic platforms that regulate the function and trafficking of crucial membrane proteins^16^. Preclinical studies indicate that GM1 modulates neuronal excitability and affects hippocampal circuitry^17^. Furthermore, gangliosides are essential for normal neural development and function^18^, and their roles in the central nervous system are complex and multifaceted^19^. Notably, GM1 has been shown to influence the transport of key neuronal components^20^, and ganglioside lipids can inhibit the aggregation of the Alzheimer’s amyloid-β peptide^21^.

Recent evidence has highlighted the therapeutic potential of GM1 ganglioside in neurodegenerative disorders. These effects have been attributed to its roles in neuronal membrane stabilization, neurotrophic signaling, and synaptic modulation. GM1 can interact with amyloid-β peptides, influence aggregation processes, and modulate neurotrophin receptor signaling pathways that are impaired during Alzheimer’s disease progression. These mechanisms suggest that GM1 may contribute to restoring neuronal integrity and functional activity under pathological conditions^22–24^.

However, the net impact of GM1 on in vivo hippocampal network activity, particularly under conditions of cholinergic deficiency that model AD circuit pathophysiology, remains poorly understood. Previous investigations into gangliosides’ effects in neurodegenerative contexts have shown potential^25,26^, but variations across experimental models and conditions persist. Moreover, the dose–response relationship of GM1’s effects on neuronal excitability and its potential state-dependency in compromised neural networks are not well characterized^27,28^.

Although neuronal hyperactivity has been reported in early Alzheimer’s disease, reduced neuronal responsiveness and impaired synaptic signaling have also been observed in specific neuronal populations and disease stages. Therefore, pharmacological modulation of neuronal activity should be interpreted relative to baseline functional deficits. Restoration of neuronal responsiveness toward physiological levels rather than simple enhancement of excitability may underlie potential therapeutic benefits, emphasizing the importance of dose-dependent investigations^4,29^.

Despite accumulating evidence regarding the neuroprotective properties of GM1, limited information is available concerning its dose-dependent electrophysiological effects on hippocampal neuronal activity in both healthy and Alzheimer-like conditions using in vivo approaches. Comparative studies across physiological and pathological conditions remain scarce^30,31^.

Importantly, increased neuronal firing in disease models should not be equated with pathological hyperexcitability without consideration of baseline network state, anesthetic conditions, and circuit context. Thus, interpreting pharmacological modulation requires careful physiological framing^32^.

In this study, we employed in vivo single-unit recordings to test the hypothesis that systemic GM1 administration enhances the spontaneous firing of hippocampal CA1 pyramidal neurons in a dose-dependent manner. We further hypothesized that this effect would be more pronounced in the compromised network of NBM-lesioned rats, reflecting a potential therapeutic selectivity of GM1 for cholinergic-deficient circuits relevant to AD.

## Results

A total of 56 rats were included in the study (7 animals per experimental group), from which 87 CA1 pyramidal neurons were recorded (mean ± SD: 2.2 ± 0.9 neurons per animal; range: 1–4). Histological analysis confirmed accurate lesion and electrode placement in all included animals (Fig. 4). All recordings were obtained under stable urethane anesthesia following a 15-min baseline period.

### GM1 increases spontaneous firing of CA1 pyramidal neurons

**Fig. 1:**
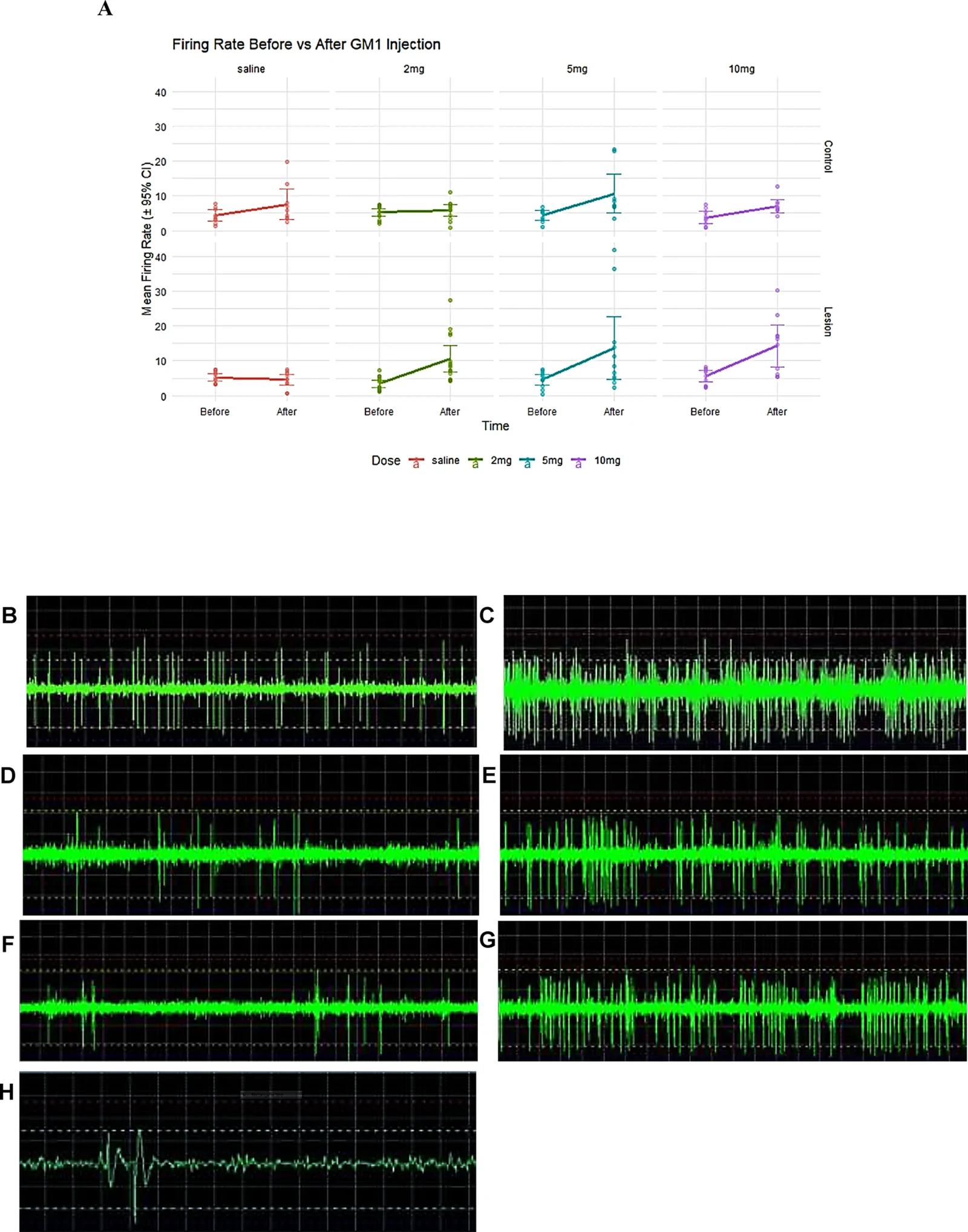
Dose-dependent effects of GM1 on CA1 pyramidal neuron firing. (**A**) Mean spontaneous firing rate (± 95% CI) before and after systemic GM1 administration in Control and NBM-lesioned rats. Individual data points are overlaid; lines connect group means. Linear mixed-effects analysis revealed significant main effect of Time and Dose × Time interaction. (**B**,** C**) Representative firing activity before and after GM1 injection (2 mg/kg); (**D, E**) Firing activity before and after GM1 injection (5 mg/kg); (**F**, **G**) Firing activity before and after GM1 injection (10 mg/kg); (**H**) Typical spike waveform of a CA1 pyramidal neuron (duration > 1.3 ms).

As shown in Fig. 1A, systemic administration of GM1 produced an overall increase in spontaneous firing rate of hippocampal CA1 pyramidal neurons compared with baseline across experimental conditions.

In control animals, GM1 induced dose-dependent changes in firing rate. The most pronounced increase was observed at 5 mg/kg (baseline: 4.36 ± 0.62 Hz; post: 10.61 ± 2.41 Hz; Δ = 6.26 ± 2.19 Hz, 95% CI [1.20, 11.31]). A moderate increase was also observed at 10 mg/kg (baseline: 3.73 ± 0.77 Hz; post: 7.03 ± 0.80 Hz; Δ = 3.30 ± 0.98 Hz, 95% CI [1.05, 5.55]). In contrast, low-dose GM1 (2 mg/kg) produced only a minimal change (Δ = 0.60 ± 0.42 Hz, 95% CI [− 0.32, 1.52]), similar to saline-treated controls (Δ = 3.27 ± 2.00 Hz, 95% CI [− 1.35, 7.89]).

### NBM lesion enhances GM1-induced increases in firing rate

In NBM-lesioned animals, GM1 produced greater increases in CA1 pyramidal neuron firing rates than in control animals (Fig. 1A, Table 1). This effect was evident across doses, particularly at lower concentrations.

**Table 1:** Effects of GM1 administration on CA1 firing rate.

Group	n (Rats)	n (neurons)	Before (Hz, Mean ± SEM)	After (Hz, Mean ± SEM)	Δ (Mean ± SEM)	95% CI of Δ
Control + 10 mg/kg	7	9	3.73 ± 0.77	7.03 ± 0.80	3.30 ± 0.98	[1.05, 5.55]
Control + 2 mg/kg	7	13	5.24 ± 0.52	5.84 ± 0.73	0.60 ± 0.42	[− 0.32, 1.52]
Control + 5 mg/kg	7	9	4.36 ± 0.62	10.61 ± 2.41	6.26 ± 2.19	[1.20, 11.31]
Control + Saline	7	9	4.29 ± 0.71	7.56 ± 1.88	3.27 ± 2.00	[− 1.35, 7.89]
Lesion + 10 mg/kg	7	10	5.50 ± 0.73	14.27 ± 2.63	8.77 ± 2.64	[2.80, 14.74]
Lesion + 2 mg/kg	7	15	3.37 ± 0.49	10.59 ± 1.73	7.22 ± 1.82	[3.33, 11.11]
Lesion + 5 mg/kg	7	11	4.56 ± 0.67	13.62 ± 4.02	9.05 ± 3.74	[0.71, 17.40]
Lesion + Saline	7	11	5.23 ± 0.49	4.61 ± 0.71	− 0.62 ± 0.39	[− 1.48, 0.25]

Data are presented as Mean ± SEM. Δ represents the change following injection. 95% confidence intervals were calculated using Student’s t distribution. To account for the nested structure of the data (multiple neurons per animal), statistical significance was assessed using linear mixed-effects models with animal ID as a random intercept (see Methods and Table 2).

At 2 mg/kg, lesioned animals showed a marked increase in firing rate (baseline: 3.37 ± 0.49 Hz; post: 10.59 ± 1.73 Hz; Δ = 7.22 ± 1.82 Hz, 95% CI [3.33, 11.11]). At 5 mg/kg, a robust increase was also observed (Δ = 9.05 ± 3.74 Hz, 95% CI [0.71, 17.40]). At 10 mg/kg, a robust increase was also observed from 5.50 ± 0.73 Hz to 14.27 ± 2.63 Hz (Δ = 8.77 ± 2.64 Hz, 95% CI [2.80, 14.74]). Saline-treated lesioned animals showed no significant change (Δ = − 0.62 ± 0.39 Hz, 95% CI [− 1.48, 0.25]).

### Distributional analysis confirms consistent rightward shift in firing rates

**Fig. 2:**
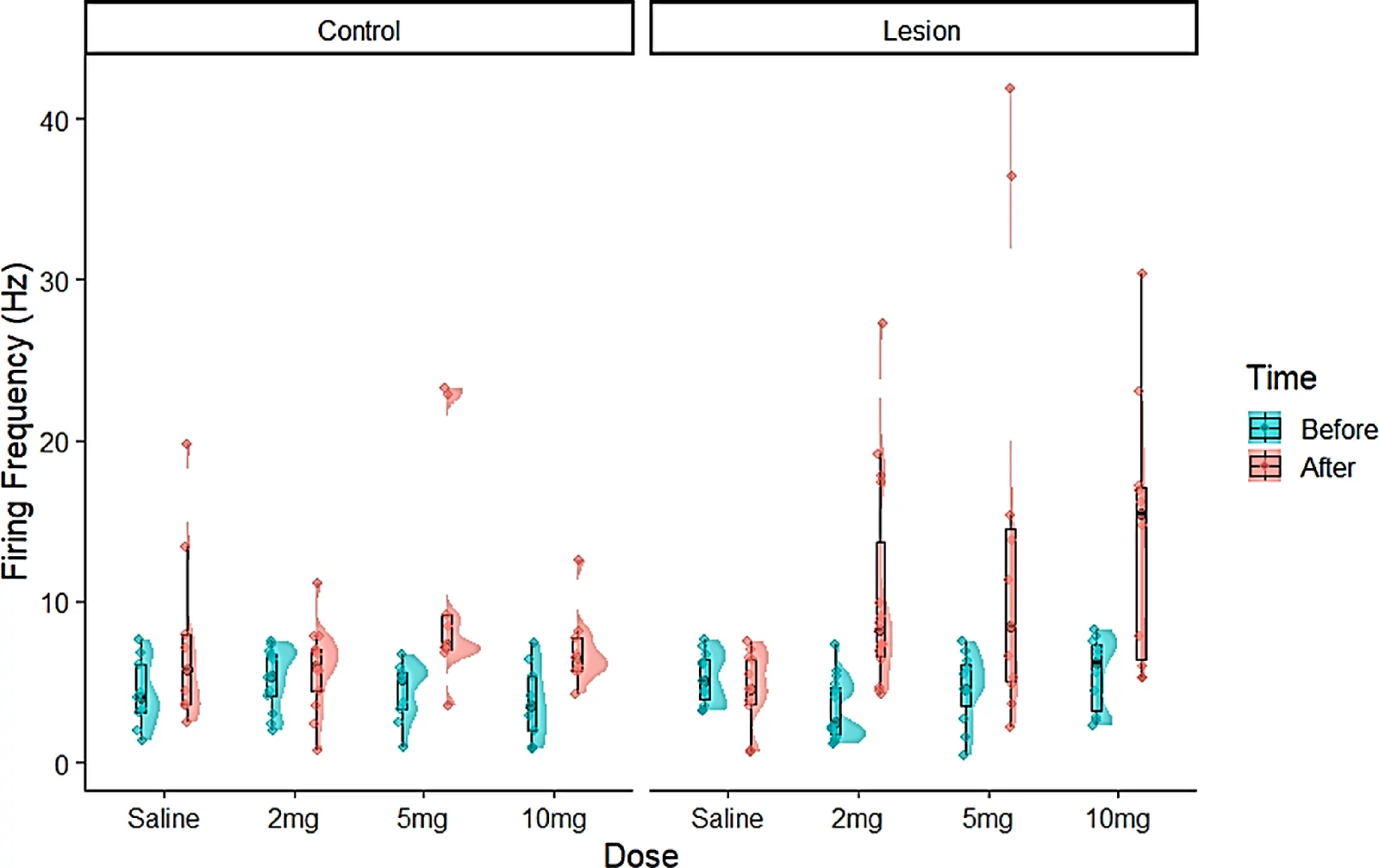
Distribution of CA1 pyramidal neuron firing rates before and after systemic GM1 administration. Raincloud plots show spontaneous firing frequency across doses (saline, 2, 5, and 10 mg/kg) in control and NBM-lesioned rats. Individual data points represent single-unit recordings; boxplots indicate median and interquartile range; half-violins depict data distribution. Linear mixed-effects analysis (Type III ANOVA with Satterthwaite approximation) revealed a significant main effect of Time and a significant Dose × Time interaction, indicating dose-dependent enhancement of firing rate following GM1 administration.

Raincloud plot analysis (Fig. 2) demonstrated a consistent rightward shift in CA1 firing rate distributions following GM1 administration across doses in both control and lesioned animals. A visually greater shift was observed in NBM-lesioned animals, particularly at 5 and 10 mg/kg.

Individual single-unit recordings confirmed that increases in firing rate were broadly distributed across neurons rather than being driven by a small subset of high responders (Fig. 2).

### Individual neuron analysis confirms dose-dependent response patterns

**Fig. 3:**
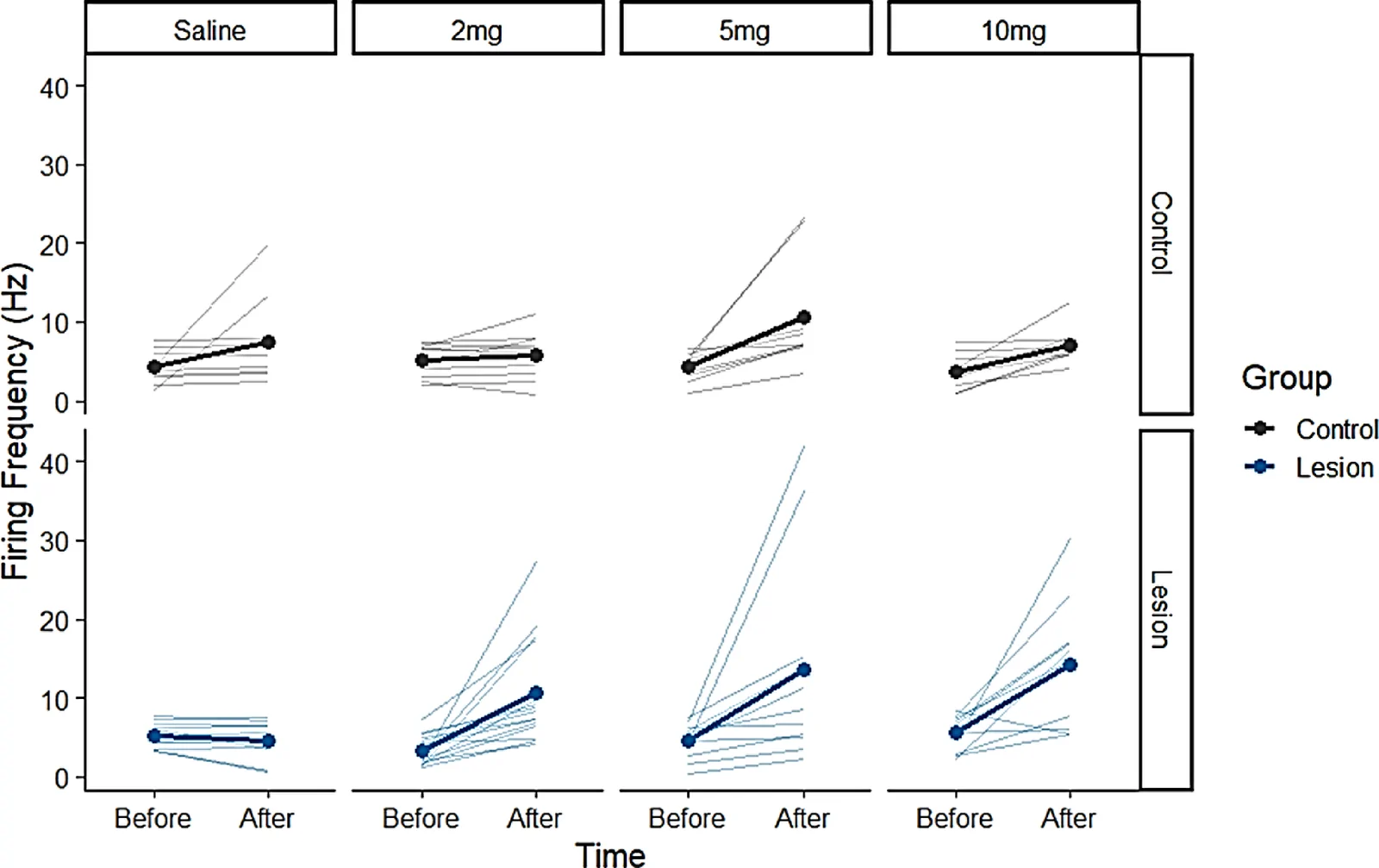
Individual neuronal trajectories before and after systemic GM1 administration. Each grey line represents a single neuronal recording. While increases in firing rate are observed across doses, lesioned animals demonstrate greater variability and larger post-injection responses at higher doses, consistent with the significant Dose × Time interaction detected in the mixed-effects model.

Analysis of individual neuronal trajectories (Fig. 3) showed that most recorded neurons exhibited an increase in firing rate following GM1 administration.

Lesioned animals displayed larger post-injection increases and greater variability at higher doses compared with controls, particularly at 5 and 10 mg/kg. These patterns were consistent across animals and reflected the overall Dose × Time interaction observed in the statistical model (Table 2).

**Table 2:** Type III linear mixed-effects ANOVA results.

Effect	F (df1, df2)	*p*-value	Partial η^2^
Lesion status	3.71 (1,79)	0.057	0.04
Dose	2.25 (3,79)	0.089	0.08
Time	41.33 (1,79)	< 0.001	0.34
Lesion Status × Dose	1.61 (3,79)	0.193	0.06
Lesion Status × Time	3.49 (1,79)	0.065	0.04
Dose × Time	3.28 (3,79)	0.025	0.11
Lesion Status × Dose × Time	2.59 (3,79)	0.051	0.09

Linear mixed-effects model with Satterthwaite approximation. Animal ID included as a random intercept.

### Linear mixed-effects model confirms significant time and dose-dependent effects

Linear mixed-effects modeling revealed a significant main effect of Time (pre vs post injection), indicating an overall increase in firing rate following GM1 administration (F_1,79_ = 41.33, *p* < 0.001, partial η^2^ = 0.34).

A significant Dose × Time interaction was also observed (F_3,79_ = 3.28, *p* = 0.025, partial η^2^ = 0.11), indicating that the magnitude of GM1-induced changes depended on dose (Table 2).

Although the main effect of Lesion Status did not reach statistical significance (F_1,79_ = 3.71, *p* = 0.057), a trend toward a Lesion × Dose × Time interaction was observed (F_3,79_ = 2.59, *p* = 0.051).

### No abnormal firing patterns observed

Across all experimental conditions, GM1-induced increases in firing rate remained within previously reported physiological ranges for CA1 pyramidal neurons under urethane anesthesia. No epileptiform discharges or abnormal burst firing patterns were observed.

Baseline firing rates did not differ substantially between control and NBM-lesioned animals prior to drug administration.

### Histological verification

**Fig. 4:**
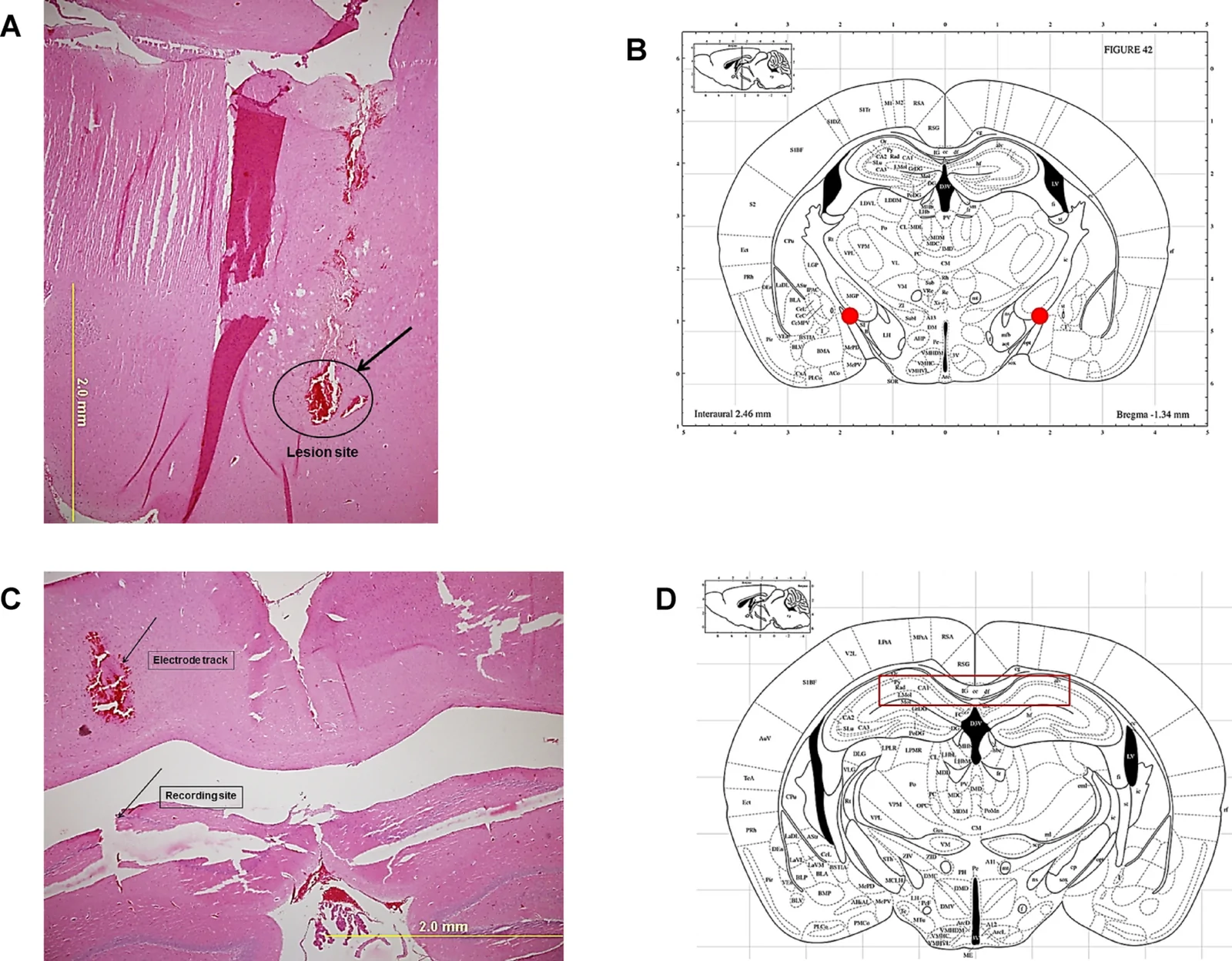
Histological verification of NBM lesions and electrode placement. (**A**) Representative H&E-stained coronal section showing the NBM lesion site (arrow). Scale bar = 2.0 mm. (**B**) Schematic diagram of a coronal brain section at Bregma − 1.3 mm showing the NBM region and lesion extent. (**C**) Representative electrode track and recording site within the hippocampal CA1 region. Scale bar = 2.0 mm. (**D**) Schematic diagram at Bregma − 3.5 mm indicating the CA1 recording area.

Histological analysis confirmed correct bilateral NBM lesions and accurate placement of recording electrodes within the CA1 pyramidal layer in all included animals (Fig. 4). Only animals with verified lesion and electrode placement were included in the final dataset.

## Discussion

The present study demonstrates that systemic administration of GM1 ganglioside enhances the spontaneous firing rate of hippocampal CA1 pyramidal neurons in a dose-dependent manner, as evidenced by a significant Dose × Time interaction (F_3,79_ = 3.28, *p* = 0.025, partial η^2^ = 0.11). Notably, a trend toward state-dependent responsiveness was observed in NBM-lesioned rats (Lesion × Dose × Time interaction, *p* = 0.051, partial η^2^ = 0.09). Together, these findings provide in vivo evidence that GM1 modulates neuronal excitability in a dose-dependent manner and may exhibit state-dependent effects under conditions of cholinergic dysfunction. Importantly, these results should be interpreted as modulation of single-neuron excitability rather than direct evidence of network-level changes.

Our observation that GM1 increased CA1 neuronal firing is consistent with previous studies describing the neurotrophic and neuroprotective properties of gangliosides. Early work has shown that GM1 can facilitate recovery of high-affinity choline uptake (HACU) following NBM lesions, suggesting preservation of cholinergic terminal function in compromised circuits^4,7,10^. Pedata and colleagues reported that NBM lesion induces a significant reduction in HACU activity in cortical regions, which was prevented by GM1 administration^4,7^. Such preservation of cholinergic markers may contribute to the enhanced neuronal responsiveness observed in the present study.

In addition, Garofalo and Cuello demonstrated that GM1 treatment can prevent structural degeneration of cholinergic neurons and maintain their neuritic architecture following cortical lesions^33^. These findings, together with our electrophysiological data, suggest that GM1 may influence both functional excitability and structural stability within cholinergic-deficient systems. In our study, the greater firing increase observed in NBM-lesioned animals compared with controls likely reflects altered cholinergic tone and increased sensitivity to membrane-level modulation.

The significant Dose × Time interaction (*p* = 0.025, partial η^2^ = 0.11) indicates that GM1 effects are concentration-dependent, consistent with its role in lipid raft organization and membrane signaling regulation. GM1 is known to integrate into lipid microdomains and modulate the function and trafficking of membrane proteins involved in neuronal excitability^16,21^. In the context of Alzheimer’s disease-related literature, ganglioside metabolism has been implicated in amyloid-β (Aβ) interactions. Ariga and colleagues reported that GM1 can bind Aβ and form complexes detected in Alzheimer’s disease brains^33^. While such interactions have been associated with aggregation processes, their functional consequences on neuronal excitability remain unclear. In the present acute in vivo setting, GM1-induced changes in firing are more likely to reflect membrane-associated modulation of ion channels or receptor function rather than amyloid-related mechanisms.

Wang and colleagues further demonstrated that GM1 can modulate γ-secretase activity via interactions with presenilin-1, thereby influencing amyloid precursor protein processing^3^. Although reductions in GM1 have been associated with reduced amyloid burden in experimental models, our findings highlight that GM1 may exert distinct effects depending on cellular and circuit state. In the present cholinergic lesion model, GM1 appears to enhance neuronal responsiveness in a state-dependent manner rather than acting as a uniform excitatory agent.

A near-significant Lesion × Dose × Time interaction (*p* = 0.051, partial η^2^ = 0.09) suggests a biologically relevant trend toward increased sensitivity to GM1 following cholinergic depletion. This may reflect altered dependence of neurons on membrane-associated signaling mechanisms in the absence of normal basal forebrain input^34^.

Ledeen and colleagues emphasized that GM1 is essential for neuronal function and that altered ganglioside composition has been observed in neurodegenerative conditions^35^. However, Alzheimer’s disease is associated with complex and region-dependent alterations in ganglioside metabolism, involving both a-series and b-series gangliosides^35^. This complexity likely contributes to variability in reported functional outcomes and highlights the importance of circuit-specific experimental approaches.

The greater responsiveness observed in lesioned animals, particularly at intermediate and high doses, suggests that GM1 may preferentially modulate circuits with reduced cholinergic tone. This state-dependent effect is consistent with the concept that pharmacological agents may exert different functional outcomes depending on baseline neuromodulatory conditions^4,29^.

Importantly, hippocampal circuit dysfunction in Alzheimer’s disease is increasingly recognized as heterogeneous rather than uniform. Busche and Konnerth have shown that hippocampal networks may exhibit both hyperactive and hypoactive states depending on disease stage and neuronal population^4^. Therefore, changes in firing rate should not be interpreted in isolation as beneficial or detrimental but rather in relation to baseline circuit state.

In this study, GM1 did not simply increase neuronal firing uniformly. In control animals, low-dose GM1 produced minimal changes, whereas in NBM-lesioned animals, even low doses resulted in pronounced increases in firing rate. This pattern suggests state-dependent modulation of neuronal responsiveness rather than nonspecific excitation.

It is important to emphasize that baseline firing rates in NBM-lesioned animals were not significantly different from controls under urethane anesthesia, consistent with a model of cholinergic hypofunction rather than intrinsic hyperexcitability. CA1 pyramidal neurons under urethane typically exhibit low spontaneous activity within a physiological range. The increases observed after GM1 administration remained within previously reported physiological limits and were not associated with epileptiform activity or abnormal bursting patterns. Therefore, these changes are interpreted as modulation of neuronal excitability within a compromised cholinergic system rather than pathological overactivation.

Methodologically, this study provides in vivo single-unit evidence for dose-dependent modulation of CA1 pyramidal neuron activity by GM1. Previous studies have primarily relied on biochemical markers, structural endpoints, or in vitro systems^1,4,7^. The present approach allows direct quantification of acute neuronal responsiveness under physiologically relevant conditions.

The observed dose-dependent effects are consistent with previous in vitro findings showing that GM1 can exert concentration-dependent neuroprotective and cellular effects^36^, suggesting that its influence on neuronal function is robust across experimental systems.

Finally, it is important to contextualize the firing rates observed at higher GM1 doses within the physiological range reported for CA1 neurons under urethane anesthesia. Reported firing rates can vary depending on experimental conditions, with values up to 15–20 Hz observed following pharmacological modulation^39,45^. In our study, both baseline and post-GM1 firing rates remained within this range, supporting the interpretation that GM1 enhances physiological excitability rather than inducing pathological hyperactivity. The absence of epileptiform discharges further supports this conclusion.

### Limitations and future directions

Several limitations of this study should be acknowledged. First, while the NBM lesion model recapitulates cholinergic hypofunction relevant to AD, it does not capture the full complexity of AD pathology, including amyloid and tau accumulation. Future studies should examine GM1’s effects in transgenic AD models (e.g., APP/PS1 or 5xFAD mice) to determine how amyloid pathology modulates GM1 responsiveness.

Second, our acute recording paradigm cannot address whether GM1’s effects are sustained with chronic administration or translate to behavioral improvement. Longitudinal studies correlating electrophysiological changes with cognitive outcomes are needed.

Third, the cellular mechanisms underlying GM1’s excitability enhancement—whether mediated by muscarinic receptors, ion channel modulation, or synaptic plasticity—require pharmacological dissection.

Fourth, **it is important to acknowledge the exclusive use of male rats as a limitation of this study**. Only male rats were used to avoid potential confounding effects of hormonal variations associated with the estrous cycle on neuronal excitability and to maintain consistency with previous electrophysiological studies in the NBM lesion model. However, given the higher incidence of Alzheimer’s disease in females and evidence of sex differences in cholinergic system function and neuronal excitability^43,44^, future investigations should include both sexes to determine whether GM1’s effects are modulated by biological sex and to enhance the translational relevance of these findings.

Fifth, the sample size, while sufficient to detect significant effects in the linear mixed-effects model, may limit the generalizability of subgroup analyses. Future studies with larger cohorts would allow more detailed examination of inter-animal variability.

## Conclusions

In summary, we provide the first in vivo evidence that systemic GM1 administration enhances hippocampal CA1 pyramidal neuron firing in a dose-dependent manner, with amplified effects under cholinergic-deficient conditions. These findings support the concept that GM1 exerts state-dependent modulation of neuronal excitability, preferentially modulating activity in compromised circuits while exerting smaller effects in healthy networks. By bridging decades of biochemical and behavioral research on GM1 with modern in vivo electrophysiology, our study advances understanding of how gangliosides might be leveraged therapeutically in Alzheimer’s disease and related dementias. The dose-dependent and lesion-selective effects we observed provide a rationale for future investigations of GM1 as a circuit-based therapeutic agent in neurodegenerative disorders.

## Methods

### Animals and ethical approval

Only male rats (8–10 weeks old, 200–220 g, n = 56) were used in this study to minimize variability associated with estrous cycle-related hormonal fluctuations and to maintain comparability with previous studies employing the NBM lesion model^37,38^. The animals were obtained from the animal breeding center of Ahvaz Jundishapur University of Medical Sciences and housed in standard polypropylene cages under controlled conditions (12 h light/dark cycle, temperature 22 ± 2 °C) with ad libitum access to food and water. All experimental procedures were approved by the Institutional Animal Care and Use Committee of Shahid Chamran University of Ahvaz and conducted in accordance with the National Institutes of Health Guide for the Care and Use of Laboratory Animals and ARRIVE guidelines.

### NBM lesion surgery and histological verification

Bilateral electrolytic lesions of the Nucleus Basalis Magnocellularis (NBM) were performed under ketamine-xylazine anesthesia (78 mg/kg and 3 mg/kg, i.p., respectively)^37,38^. Rats were positioned in a stereotaxic apparatus (Stoelting, USA) with the skull leveled. Lesions were made by passing constant anodal current (0.5 mA for 3 s) through a Teflon-insulated stainless steel electrode (150 μm tip diameter) targeted at coordinates relative to bregma: AP − 1.3 mm, ML ± 2.8 mm, DV − 7.6 mm ^37^. Figure 3. Individual neuronal trajectories before and after systemic GM1 administration. Each grey line represents a single neuronal recording. While increases in firing rate are observed across doses, lesioned animals demonstrate greater variability and larger post-injection responses at higher doses, consistent with the significant Dose × Time interaction detected in the mixed-effects model. After electrophysiological recordings, rats were transcardially perfused with 4% paraformaldehyde. Brains were sectioned at 50 μm and stained with hematoxylin and eosin (H&E) for verification of lesion placement and electrode tracks by an observer blinded to experimental groups (Fig. 4).

### Drug preparation and administration

GM1 ganglioside (Sigma-Aldrich, USA; derived from bovine brain) was freshly dissolved in sterile 0.9% saline and administered intraperitoneally at doses of 2, 5, or 10 mg/kg (injection volume: 1 ml/kg). Control animals received equivalent volumes of sterile saline. All injections were performed after obtaining stable 15-min baseline recordings.

### In vivo single-unit recording

Rats were anesthetized with urethane (1.5 g/kg, i.p.), selected due to its minimal suppression of hippocampal synaptic transmission and preservation of spontaneous neuronal firing patterns compared with other anesthetic agents, with supplemental doses (0.1 g/kg) as needed to maintain surgical anesthesia. Following tracheostomy, animals were positioned in a stereotaxic frame with body temperature maintained at 37.0 ± 0.5 °C. A craniotomy was performed over the dorsal hippocampus (AP − 3.5 mm, ML − 2.2 mm from bregma). Single-unit activity was recorded extracellularly from CA1 pyramidal neurons using parylene-coated tungsten microelectrodes (5 MΩ impedance, FHC, USA) advanced to approximately 2.4 mm ventral to the brain surface. CA1 pyramidal neurons were identified based on characteristic low spontaneous firing rates (≤ 8 spikes/s) and broad action potential waveforms (> 1.3 ms)^39^. Neural signals were amplified (10,000 ×), band-pass filtered (300–5000 Hz), and digitized at 40 kHz using a CED Power1401 system (Cambridge Electronic Design, UK).

### Data analysis and response criteria

Offline spike sorting was performed using Spike2 software (Cambridge Electronic Design, UK) with Principal Component Analysis followed by T-distribution Expectation Maximization to isolate single units. Firing rates were calculated in consecutive 1-min bins. A neuron was considered responsive if its firing rate exceeded (excitation) or fell below (inhibition) the baseline mean ± 2 standard deviations for at least three consecutive bins. Response latency was defined as the time from injection to the first significant bin, and duration as the total time the response remained significant.

### Statistical analysis

All statistical analyses were performed using R (version 4.2.1; R Foundation for Statistical Computing, Vienna, Austria). Data are presented as mean ± SEM, with statistical significance set at *p* < 0.05. Normality was confirmed using the Shapiro–Wilk test.

To account for the nested structure of the data (multiple neurons per animal) and repeated-measures design, a linear mixed-effects model (LMM) was fitted using the lme4 package^40^. The model included firing rate as the dependent variable, with Lesion Status (control vs. lesioned), Dose (saline, 2, 5, 10 mg/kg), and Time (pre vs. post injection) as fixed factors, with Time treated as a repeated-measures factor within neurons and Animal ID included as a random intercept. Type III ANOVA with Satterthwaite approximation (lmerTest package) was used to obtain F statistics and *p* values^41^. Effect sizes are reported as partial η^2^. Where significant interactions were detected (Dose × Time, *p* = 0.025), post-hoc comparisons were performed using estimated marginal means with Tukey correction (emmeans package)^42^. Change scores (Δ = post − pre) were calculated for descriptive purposes, with within-group comparisons using paired t-tests and between-group comparisons using independent t-tests with Welch’s correction where appropriate. Sample size was determined based on previous electrophysiological studies employing similar experimental designs rather than formal a priori power calculations.

### Consideration of biological replication

Because multiple neurons were recorded from individual animals, we accounted for the nested data structure by including Animal ID as a random intercept in the linear mixed-effects model. This approach prevents pseudoreplication and ensures that statistical inference reflects biological replication at the level of the animal rather than the neuron. On average, 2.2 ± 0.9 neurons were recorded per animal, and neurons were distributed across animals within each experimental group. Thus, the statistical unit for fixed effects inference was appropriately modeled at the animal level while preserving within-animal variability. The distribution of neurons across animals did not differ systematically between experimental groups.

### Ethics declaration

All experiments were approved by the Institutional Animal Care and Use Committee of Shahid Chamran University of Ahvaz and conducted in accordance with ARRIVE guidelines and the National Institutes of Health Guide for the Care and Use of Laboratory Animals.

## Data Availability

The data that support the findings of this study are available from the corresponding author upon reasonable request.
